# ROECSG report: Real‐world implementation patterns and perceptions of artificial intelligence in radiation oncology

**DOI:** 10.1002/acm2.70871

**Published:** 2026-10-21

**Authors:** James J Sohn, Cory Knill, Elizabeth Bossart, Samuel Chen, Rohan K Patel, Kevin Du, Leah M Katz

**Affiliations:** ^1^ Department of Radiation Medicine University of Wisconsin Madison Wisconsin USA; ^2^ Department of Radiation Oncology University of Michigan Ann Arbor Michigan USA; ^3^ Department of Radiation Oncology University of Miami Miller School of Medicine Miami Florida USA; ^4^ Department of Radiation Oncology University of Alabama at Birmingham Birmingham Alabama USA; ^5^ Department of Radiation Oncology Medical College of Wisconsin Milwaukee Wisconsin USA; ^6^ Department of Therapeutic Radiology Yale University New Haven Connecticut USA

**Keywords:** artificial intelligence, implementation science, machine learning, radiation oncology, survey research

## Abstract

**Purpose:**

While international surveys have examined radiation oncology professionals’ attitudes toward artificial intelligence (AI) and documented generally low early utilization, actual implementation patterns and the depth of integration among engaged practitioners remain unclear. This multi‐institutional survey aims to characterize current AI implementation, identify critical barriers to optimization, and evaluate the psychological factors driving clinical trust.

**Methods and materials:**

A de‐identified survey covering six domains was developed by a multi‐institutional working group and distributed via a widely subscribed medical physics listserv and at the 2025 American Association of Physicists in Medicine (AAPM) Annual Meeting. The instrument assessed adoption rates, specific workflow applications, and user perceptions (trust and understanding) using 6‐point Likert scales to reduce central tendency bias. Quantitative data were analyzed using Pearson correlation coefficients and Welch's two‐sample *t*‐tests, while qualitative responses underwent inductive thematic analysis (κ = 0.78).

**Results:**

In this convenience sample of 80 respondents (46.3% radiation oncologists, 37.5% medical physicists), 85.0% (68/80) reported current AI use. Adoption is heavily concentrated in auto‐contouring (88.2% of AI users), whereas diagnosis/prognostication (14.7%) and treatment planning (23.5%) remain limited. Most users (60.3%) employ AI for task automation without influencing clinical judgment. Trust in AI (3.55 ± 1.02) correlates positively with perceived time savings (*r* = 0.57), clarity of use (*r* = 0.54), and technical understanding (*r* = 0.51, all *p* < 0.001), with no significant difference among these correlations (Williams test, both *p* > 0.59). Notably, self‐rated understanding averaged near the scale midpoint (3.44 ± 1.22), yet 88.8% welcomed greater AI integration. Qualitative analysis identified transparency (38%) and validation protocols (31%) as the primary barriers to deeper implementation.

**Conclusions:**

AI use was common within this sample (85.0%), although self‐selection likely inflates this figure relative to the broader field. However, implementation remains “broad but shallow,” focused on workflow automation rather than clinical intelligence. The gap between enthusiasm and understanding necessitates a shift toward active institutional governance and standardized AI‐literacy education.

## 1 INTRODUCTION

Artificial intelligence (AI) is rapidly transitioning from an experimental tool to an operational necessity in radiation oncology, with applications spanning auto‐contouring, treatment planning optimization, quality assurance, and clinical decision support. This shift is accelerated by mounting clinical pressures; 2023 American Society for Radiation Oncology (ASTRO) workforce data indicate that 93% of practitioners report staff shortages, with 53% acknowledging treatment delays.^1^ While earlier international surveys documented generally low AI utilization alongside cautious optimism and notable educational gaps among radiation oncology professionals,^2^, ^3^, ^4^ the field has likely reached a new inflection point. Regulatory and implementation frameworks have also matured, with the 2024 American Association of Physicists in Medicine (AAPM)‐European Society for Radiotherapy and Oncology (ESTRO) joint guideline now providing specific implementation frameworks for radiation oncology^5^ and standardized reporting tools such as Consolidated Standards of Reporting Trials‐Artificial Intelligence (CONSORT‐AI) offering structured approaches for clinical validation.^6^

This study characterizes AI use within a convenience sample of radiation oncology professionals recruited through a national listserv and a professional meeting. By moving beyond general attitudes to analyze implementation depth, trust formation, and barrier characterization, we aim to define the current boundaries of AI integration and identify the requirements for advancing toward “treatment planning at the speed of thought.”

## 2 METHODS

The Radiation Oncology Education Collaborative Study Group (ROECSG) AI Working Group developed a survey instrument through iterative refinement among seven radiation oncology professionals across six institutions. The instrument covered six domains (demographics, adoption status, implementation depth, perceived utility, user perceptions, and barriers) and employed branching logic, presenting respondents who reported no current AI use with a future‐adoption pathway in place of the AI‐application items. The complete instrument is provided in Supplementary Material 1. Core perception items (trust, understanding, perceived utility) used a 6‐point Likert scale without a neutral midpoint to encourage directional responses; a general attitudinal item regarding willingness to expand AI use employed a 5‐point scale including a neutral option. Likert items were treated as interval‐level for correlation analysis. The survey underwent pilot testing with 12 practitioners to ensure clarity and appropriate response burden (estimated 8–10 min). The survey was distributed through the Wayne State University Medical Physics listserv and via QR codes displayed at the 2025 AAPM Annual Meeting. Recruitment was anonymous and based on voluntary participation; because an open, anonymous link was used, a response rate cannot be calculated, and the sample should be regarded as a convenience sample. No survey developers or pilot testers were among the tallied respondents. The survey was open from July 2025 through November 2025 with no incentives. Participation was voluntary and anonymous, with implied consent indicated by survey completion.

Descriptive statistics characterized distributions and response patterns. Pearson correlation coefficients examined relationships between Likert‐scale variables, with Welch's two‐sample *t*‐tests comparing continuous variables between users and non‐users. Qualitative responses underwent inductive thematic analysis following ESTRO 2024 standards for survey research;^7^ two investigators independently coded responses with disagreements resolved through consensus (Cohen's κ = 0.78). Although convenience sampling precluded a formal power calculation, the achieved sample (*N* = 80) followed recent methodological guidance.^8^ Responses were screened for duplicate entries using respondent‐provided identifiers; one near‐simultaneous duplicate submission was identified and removed, yielding a final analytic sample of 80. The survey used branching logic: respondents reporting no current AI use were directed to a future‐adoption question and did not receive items on current AI applications. Item‐level response counts therefore reflect this conditional design rather than missing data; all 80 respondents completed the core perception items. Item‐level response completeness and the branching structure are detailed in Supplementary Material 2. All analyses were performed using Python 3.12 with SciPy and Pandas. Respondents were asked for a name and institution solely to screen for duplicate submissions; these identifiers were removed prior to analysis and are not reported. The study was exempt from institutional review board approval as minimal‐risk, de‐identified professional survey research.

## 3 RESULTS

### 3.1 Adoption and workflow distribution

The respondent cohort (*N* = 80) reflected the interdisciplinary nature of the field: 37 radiation oncologists (46.3%), 30 medical physicists (37.5%), 2 residents/students (2.5%), and 11 others including dosimetrists and radiation therapists (13.8%). The reported AI adoption rate was 85.0% (68/80), higher than in earlier international surveys, which documented generally low utilization (Table 1). Among non‐users (15.0%, 12/80), 83.3% expressed intent to adopt within the next year.

**TABLE 1:** Comparative summary of international surveys on AI in radiation oncology (2020–2025).

Cohort (Year)	Design/Focus	Key findings	Reference
Current Study (Engaged US, 2025)	Multi‐domain survey	85.0% current AI use; auto‐contouring dominant; integration broad but shallow	Present study
Canada (2021)	Perceptions survey	Attitudes of four professional groups; education prioritized	Wong et al.^4^
Australia (2020)	Automation perceptions	Cautiously optimistic; concern over skill loss	Batumalai et al.^3^
New Zealand (2021)	Adoption‐likelihood survey	AI usage low; high willingness to adopt	Mugabe^2^

The higher current AI use in the present study (85.0%) likely reflects selection of engaged practitioners at professional meetings and listservs rather than general population sampling. Direct comparisons should be interpreted cautiously given methodological differences.

Among AI users (*n* = 68), auto‐contouring dominated at 88.2% (60/68), consistent with established standards for normal tissue delineation^9^ and the technical maturity of commercially available solutions (Figure 1). Applications involving clinical judgment, such as diagnosis/prognostication (14.7%, 10/68) and prescribing support (8.8%), showed substantially lower penetration. Notably, 11.8% of AI users (8/68) reported using consumer AI tools (e.g., ChatGPT ^10^) for clinical documentation.

**FIGURE 1:**
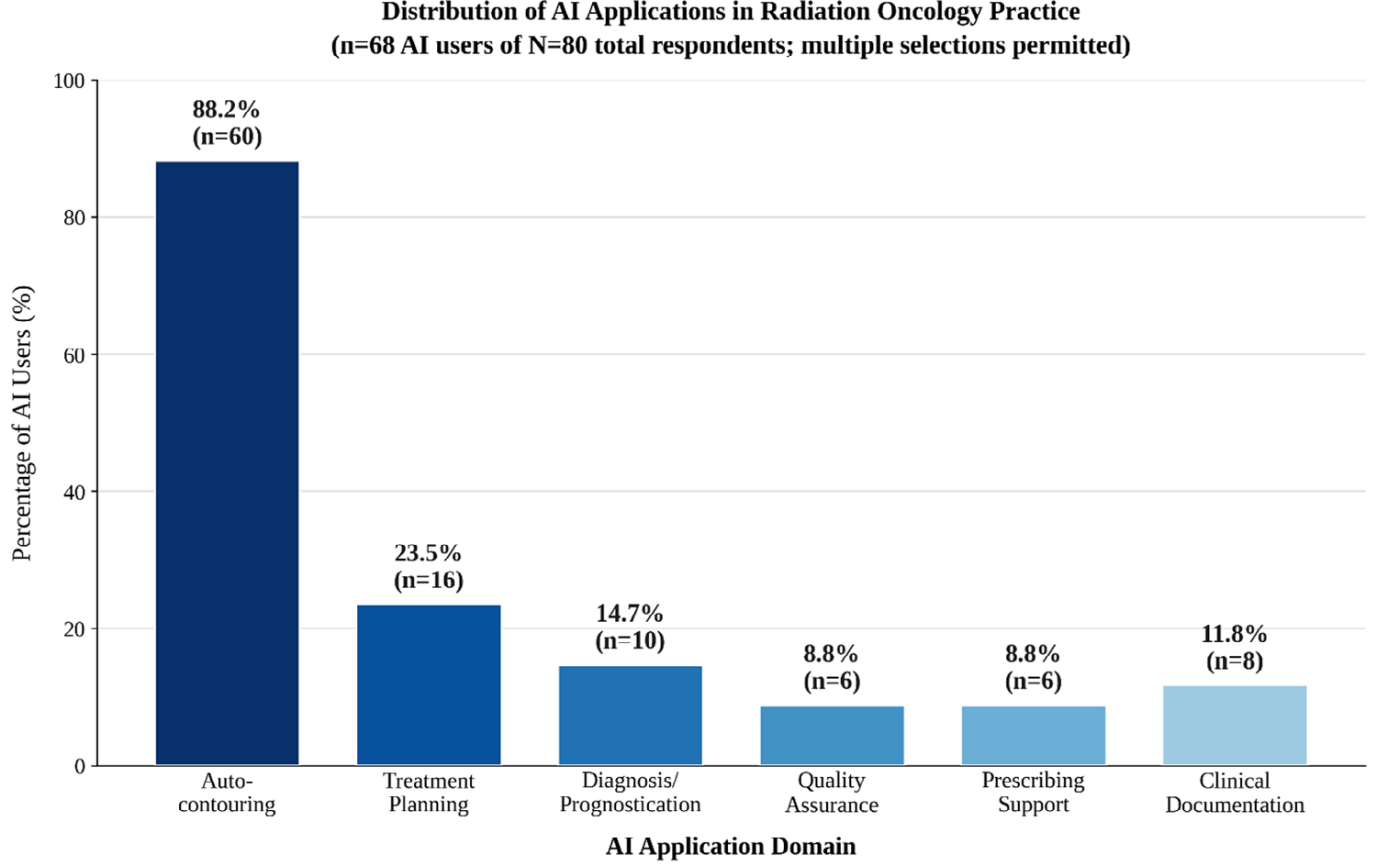
Distribution of AI applications in radiation oncology practice (*n* = 68 AI users of *N* = 80 total respondents; multiple selections permitted). Auto‐contouring dominates at 88.2% (60/68), reflecting the technical maturity of commercial segmentation tools. Applications requiring clinical judgment, namely treatment planning (23.5%) and diagnosis/prognostication (14.7%), showed substantially lower adoption. Of note, 11.8% reported using consumer AI tools (e.g., ChatGPT) for clinical documentation.

### 3.2 Implementation depth

Despite high adoption, 60.3% of AI users (41/68) characterized their implementation as “task automation only”: efficiency gains without influencing clinical judgment (Figure 2A). Only 23.5% (16/68) reported AI informing clinical management, and merely 4.4% (3/68) described AI as driving clinical decisions. A separate 11.8% (8/68) were unsure of AI's role in their decision‐making processes. This pattern suggests that current integration is “broad but shallow,” capturing efficiency but lacking depth in clinical augmentation.

**FIGURE 2:**
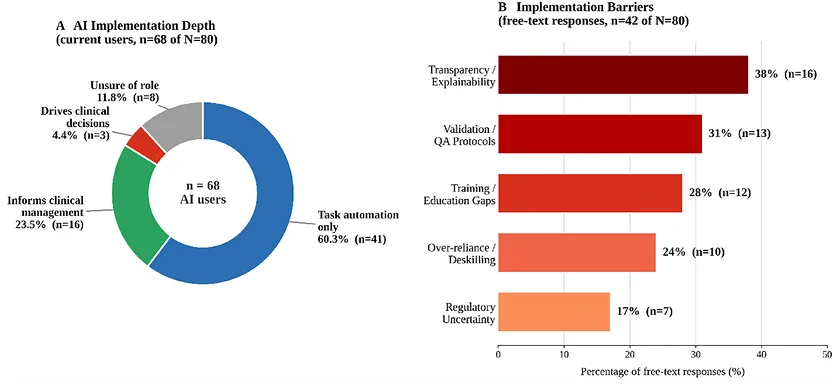
Implementation characteristics. (A) AI implementation depth among current users (*n* = 68 of *N* = 80 respondents): 60.3% (41/68) employ AI for task automation only, 23.5% (16/68) report AI informing clinical management, and only 4.4% (3/68) describe AI as driving clinical decisions. (B) Implementation barriers from thematic analysis of free‐text responses (*n* = 42 of 80 respondents; themes non‐mutually‐exclusive): transparency/explainability (*n* = 16, 38%), validation protocols (*n* = 13, 31%), training gaps (*n* = 12, 28%), over‐reliance concerns (*n* = 10, 24%), and regulatory uncertainty (*n* = 7, 17%).

### 3.3 The trust‐understanding gap

A key finding is that clinical trust is built on experiential utility rather than technical comprehension. Across all respondents (*N* = 80), self‐reported understanding of AI development averaged 3.44 ± 1.22, near the scale midpoint, while trust scored 3.55 ± 1.02. Trust correlated positively and at similar magnitudes with time savings (*r* = 0.57), clarity of use (*r* = 0.54), and understanding (*r* = 0.51, all *p* < 0.001); pairwise comparisons of these dependent correlations were not statistically significant (Williams test, both *p* > 0.59) (Figure 3). Notably, 88.8% of respondents welcomed greater AI integration and only 2.5% were opposed, indicating that enthusiasm for AI outpaces self‐assessed understanding. Compared with non‐users, current AI users reported higher perceived time savings, clarity of intended use, and workflow integration (all *p* < 0.05, Welch's *t*‐test), whereas trust and understanding did not differ between groups (p≥0.38).

**FIGURE 3:**
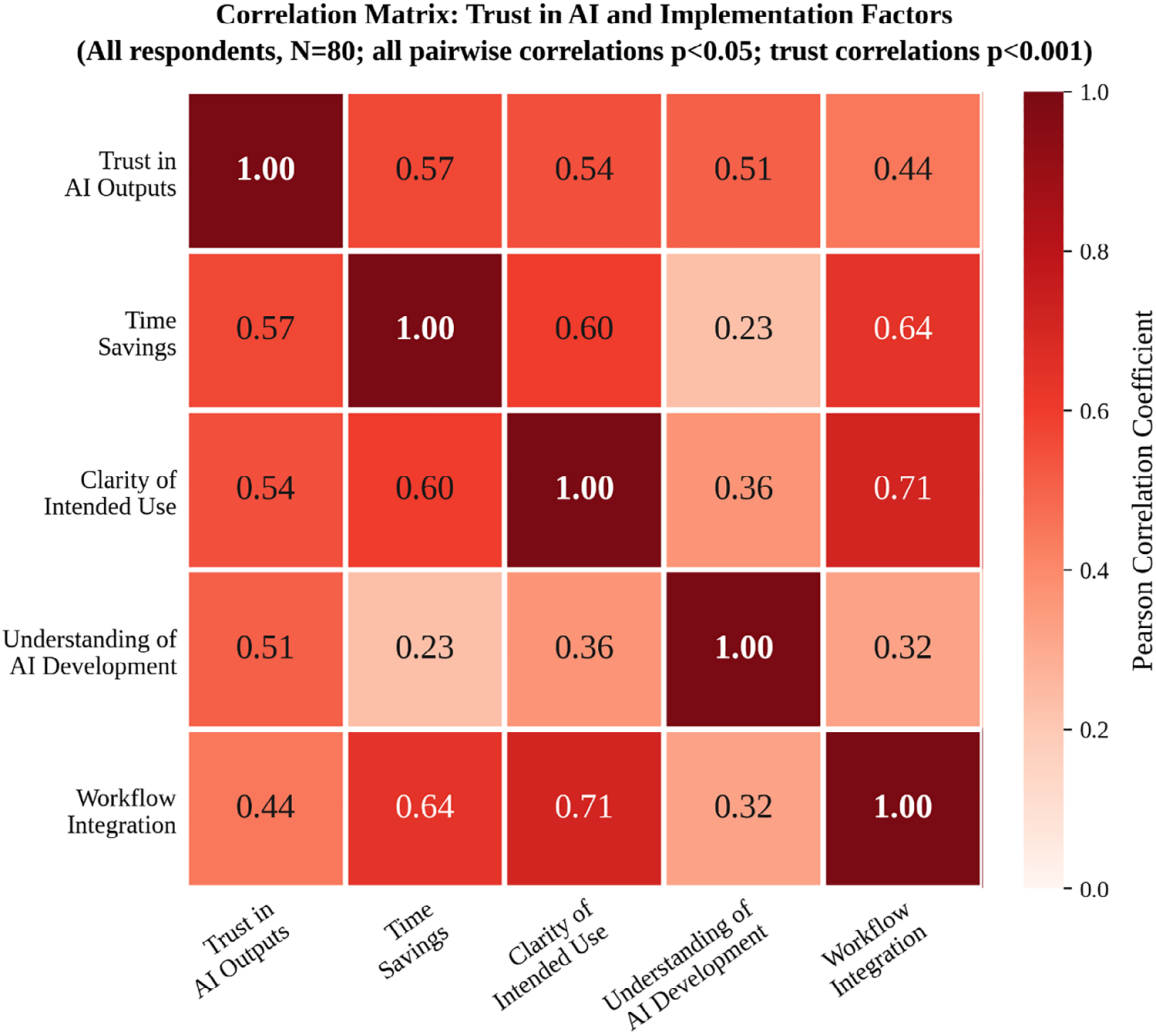
Correlation matrix of user perception variables (all respondents, *N* = 80; all pairwise correlations *p* < 0.05; the three correlations with trust all *p* < 0.001). Trust in AI correlated with time savings (*r* = 0.57), clarity of use (*r* = 0.54), and self‐reported understanding (*r* = 0.51) at similar magnitudes. This suggests trust formation is driven by experiential benefit rather than technical comprehension.

### 3.4 Qualitative barriers

Thematic analysis of free‐text responses (*n* = 42 of 80 respondents) identified five interconnected, non‐exclusive barrier categories (Figure 2B). Deidentified free‐text responses are provided in Supplementary Material 2. Transparency and explainability emerged most frequently (*n* = 16, 38%), with practitioners emphasizing the need for algorithmic interpretability. Validation and quality assurance (QA) protocol concerns (*n* = 13, 31%) focused on establishing appropriate benchmarks for continuously learning systems. Training and education gaps (*n* = 12, 28%) included specific concerns about trainees accepting AI outputs without critical evaluation. Over‐reliance and deskilling risks (*n* = 10, 24%) centered on potential skill atrophy, and regulatory uncertainty (*n* = 7, 17%) involved liability and reimbursement questions.

## 4 DISCUSSION

Within this sample, AI use was common (85.0%) but functionally concentrated in auto‐contouring, suggesting that respondents currently view AI primarily as a productivity tool rather than a clinical partner. The high rate likely reflects selection bias inherent in conference attendance and listserv participation; these individuals represent early adopters whose patterns may preview broader field trajectories.

Trust correlated with perceived time savings (*r* = 0.57) and self‐reported understanding (*r* = 0.51) at comparable magnitudes (Williams test, *p* > 0.59). Whether the field's reliance on experiential utility reflects a risk of automation bias cannot be determined from these data and warrants further study. As AI becomes more reliable for routine tasks, clinicians may develop experiential confidence that inadequately prepares them for system failures in rare edge cases. Furthermore, the usage of consumer‐grade large language models (LLMs), reported by 11.8% of AI users, for clinical tasks without institutional oversight raises important governance and policy considerations regarding appropriate validation and clinical‐use boundaries for non‐medical‐grade tools.^11^

To safely advance toward same‐day workflows enabled by “treatment planning at the speed of thought,” the field must move from passive tool adoption to active institutional governance. This includes adopting the 2024 AAPM‐ESTRO implementation guidelines for local AI validation^5^ and integrating AI literacy into residency training curricula. This study has important limitations. The sample was self‐selected and recruited through voluntary, anonymous channels, precluding calculation of a response rate and limiting generalizability; reported adoption is likely inflated relative to the broader field. The modest sample size (*N* = 80) and cross‐sectional design further constrain interpretation in a rapidly evolving landscape. Adoption patterns may also shift rapidly over short time horizons as commercially available AI tools continue to expand, potentially limiting long‐term generalizability.

## 5 CONCLUSIONS

Within this convenience sample of radiation oncology professionals recruited through a professional meeting and a listserv, 85.0% reported current AI use. Because of self‐selection inherent in voluntary, anonymous recruitment, this figure should be interpreted as descriptive of the sample rather than as an estimate of field‐wide prevalence. However, the transition from task automation to clinical intelligence may be hindered by a “trust‐understanding gap”: practitioners trust what works efficiently but do not fully comprehend the technology they employ. As AI evolves from automation toward clinical augmentation, future efforts must prioritize standardized validation, transparent reporting frameworks,^6^ and systematic education to ensure that efficiency gains do not compromise clinical rigor.s

## AUTHOR CONTRIBUTIONS

All authors contributed to the study design. J.J.S., C.K., and E.B. collected the data. J.J.S. performed data analysis and drafted the manuscript. S.C., R.K.P., K.D., and L.M.K. critically reviewed and edited the manuscript.

## CONFLICT OF INTEREST STATEMENT

The authors declare no conflicts of interest.

## ETHICS STATEMENT

IRB approval was not required as this study involved a simple survey without patient data.

## Supporting information



Supporting Information

Supporting Information

## Data Availability

All resulting data are provided in the supplementary document or available from the corresponding author upon reasonable request.
