# Supplementary material for: ROECSG report: Real‐world implementation patterns and perceptions of artificial intelligence in radiation oncology
Source: J Appl Clin Med Phys. 2026 Oct 21;27(11):e70871. doi: 10.1002/acm2.70871 (PMC13686580; doi:10.1002/acm2.70871)
Supplement: Supplementary file 1 [file ACM2-27-e70871-s001.docx]

**Supplementary Document 1**

**Survey Instrument: ROECSG Clinical AI Survey**

Self-administered, anonymous instrument delivered via Microsoft Forms across three sequential pages. The instrument used conditional branching: the response to the screening question on current AI use (Q4) determined which subsequent items each respondent received. On submission, name and institution were collected for duplicate screening only and were removed before analysis.

***Branching logic (Q4):***

*• If “Yes” (current AI user) → routed to Q5–Q6 (AI applications and decision contribution), then to Q7.*

*• If “No” (non-user) → routed to Q5alt (future-adoption intent), skipping Q5–Q6, then to Q7.*

**Page 1 of 3 – Tell us about yourself**

Presented to all respondents.

Q1. What is your full name?

Q2. What is the name of your institution?

Q3. What is your role in your home institution?

**Page 2 of 3 – Practice-Pattern Inquiry**

Presented to all respondents (screening / branch point).

Q4. Are Artificial Intelligence (AI) tools currently used in your clinical practice?

- Yes -> go to Q5 (AI-user path)
- No -> go to Q5alt (non-user path)

***AI-User Path – shown only if Q4 = “Yes”***

Q5. In which parts of the radiation oncology workflow is AI used? (select all that apply)

□ Diagnosis / Prognostication

□ Prescribing treatment

□ Simulation

□ Contouring

□ Treatment Planning

□ Quality Assurance

□ Response Assessment

□ Follow-up

□ Other (free text)

Q6. How significantly does the AI tool contribute to clinical decision-making? (select the highest level that applies)

- It autonomously diagnoses or prescribes treatment
- It drives clinical management
- It informs clinical management
- It performs a non-decision-support function
- Unsure

***Non-User Path – shown only if Q4 = “No”***

Q5alt. Do you hope to incorporate AI tools into your clinical practice in the future?

- Yes
- No

***Path Converge – Q7 shown to all respondents***

Q7. Please indicate your level of agreement with the following statement

|  | Strongly Agree | Agree | Neutral | Disagree | Strongly Disagree |
| --- | --- | --- | --- | --- | --- |
| “I would welcome greater integration of AI tools into my daily clinical workflow.” |  |  |  |  |  |

**Page 3 of 3 – AI Attitudes Inquiry**

Presented to all respondents.

Q8. Please rate the following aspects of the AI tool(s) you use (1 = None at all, 6 = Extremely High):

|  | 1 | 2 | 3 | 4 | 5 | 6 |
| --- | --- | --- | --- | --- | --- | --- |
| Your understanding of how the AI tool was developed |  |  |  |  |  |  |
| Your trust in the AI tool’s outputs |  |  |  |  |  |  |
| The amount of time it saves you or your team |  |  |  |  |  |  |
| The clarity of the tool’s intended use and limitations |  |  |  |  |  |  |
| Its integration into existing clinical workflows |  |  |  |  |  |  |

Q9. What AI tools or vendors are you currently using (if known)?

Q10. Do you have any comments or concerns about the use of AI in clinical practice?

*Note. Because of the Q4 branch, current AI users received Q5–Q6 (not Q5alt), whereas non-users received Q5alt (not Q5–Q6); all respondents received Q1–Q4 and Q7–Q10. Item numbering is editorial and reflects order of presentation. Likert response options are shown as open circles (○) to indicate single-selection radio inputs as they appeared to respondents.*
