# Supplementary material for: ROECSG report: Real‐world implementation patterns and perceptions of artificial intelligence in radiation oncology
Source: J Appl Clin Med Phys. 2026 Oct 21;27(11):e70871. doi: 10.1002/acm2.70871 (PMC13686580; doi:10.1002/acm2.70871)
Supplement: Supplementary file 2 [file ACM2-27-e70871-s002.docx]

**Supplementary Material 2**

**Data Completeness and Deidentified Free-Text Responses**

This supplement reports item-level response completeness (Part A) and the deidentified free-text responses underlying the qualitative analysis (Part B). Consistent with the branching logic shown in Supplementary Material 1, the screening question on current AI use (Q4) routed respondents to one of two paths: AI users (Q4 = Yes; n=68) received Q5–Q6, whereas non-users (Q4 = No; n=12) received the future-adoption item (Q5alt) in their place. Item-level counts therefore reflect this conditional design rather than missing data. All 80 respondents completed every core perception item.

**Part A. Item-Level Response Completeness (N = 80)**

| **Item** | **Question (abbreviated)** | **Eligible respondents** | **n** | **Completion** |
| --- | --- | --- | --- | --- |
| Q1 | Full name | All respondents | 80 | 100% |
| Q2 | Institution name | All respondents | 80 | 100% |
| Q3 | Role at home institution | All respondents | 80 | 100% |
| Q4 | AI currently used in practice? (Yes/No) — branch point | All respondents | 80 | 100% |
| Q5 | Workflow areas where AI is used (select all) — AI-user path | Q4 = Yes (AI users) | 68 | 100% of users |
| Q6 | AI contribution to clinical decision-making — AI-user path | Q4 = Yes (AI users) | 68 | 100% of users |
| Q5alt | Future-adoption intent — non-user path | Q4 = No (non-users) | 12 | 100% of non-users |
| Q7 | Agreement: welcome greater AI integration | All respondents | 80 | 100% |
| Q8a | Rating: understanding of AI development | All respondents | 80 | 100% |
| Q8b | Rating: trust in AI outputs | All respondents | 80 | 100% |
| Q8c | Rating: time savings | All respondents | 80 | 100% |
| Q8d | Rating: clarity of intended use | All respondents | 80 | 100% |
| Q8e | Rating: workflow integration | All respondents | 80 | 100% |
| Q9 | AI tools/vendors used (free text, optional) | Optional | 55 | 68.8% |
| Q10 | Comments/concerns (free text, optional) | Optional | 42 | 52.5% |

*Note. Q5 and Q6 were presented only to the 68 respondents on the AI-user path (Q4 = Yes); both were completed by all 68 (100%). The 12 non-users (Q4 = No) were instead routed to Q5alt (future-adoption intent), which all 12 completed. All respondents received Q1–Q4 and Q7–Q10. Q9 and Q10 were optional free-text fields. One duplicate submission was identified and removed.*

**Part B. Deidentified Free-Text Responses**

**B.1 AI tools and vendors in use (Q9, aggregate)**

Of the 55 respondents (68.8%) who described the AI tools or vendors in use, 38 referenced a named commercial product, 4 referenced in-house or self-developed tools, 7 referenced a general-purpose large language model, and 12 described tools only generically (e.g., “auto-contouring”) without naming a product. Categories are non-exclusive. To preserve respondent and institutional confidentiality, individual product names are not reported.

**B.2 Comments and concerns regarding AI in clinical practice (Q10, verbatim, deidentified)**

*All 42 free-text comments are reproduced below verbatim, as written by respondents. These responses form the basis of the five barrier themes reported in the main text (transparency/explainability, validation/QA protocols, training/education gaps, over-reliance/deskilling, and regulatory uncertainty).*

**R1.** Ability to have transparency to end user. Over reliance on this to lose clinical decision making.

**R2.** Current auditors or AI output have the knowledge to understand when AI has given an incorrect solution. There is concern about newer professionals believing AI doesn’t hallucinate as often as it does.

**R3.** No

**R4.** Loss of practice and knowledge through continued use of automation. Reliance on the tool may get in the way of new users learning the skill by hand.

**R5.** I think there's a lot of potential to improve clinical practice with AI; however, I think the industry needs to proceed carefully. AI, in general, is a black box to most people, and it won't take long after implementation that people will start to implicitly trust it, assuming it gives good results. This can lead to a lack of critical evaluation and accountability (I already see this with simple treatment planning scripts).

**R6.** It seems the important thing is to always check the AI outputs. It is really great for ideas and creative endeavors, but we need to be careful about clinical decisions based on AI. AI can hallucinate and sound very convincing, even when it is very wrong.

**R7.** How much of our workload should be done through AI? Specifically in research, how much should it be used to guide, interpret and present research? Especially if AI can potentially help in efficiency and data analysis, should it be used from an ethical and independent research point of view?

**R8.** The treatment planning task is very appropriate for AI. Contouring also. Checking contouring, example GTV boders outside the PTV, numbering of multiple GTVs, beam placement, etc.

**R9.** Still seems to require a lot of user intervention because it makes many errors that we now have to catch

**R10.** Concerned about people trusting AI too much and not being diligent in the QA of these AI tools. We need human interventions for each process. Also concerned about future generations relying too heavily on AI and losing knowledge and skills that AI can offer for us.

**R11.** The biggest issue is just getting hospital IT/IS security team to allow new applications to be installed. Issue with Limbus is that it must be installed on a single desktop computer and does not use a cloud database. If multiple users are using the same PC, it can be difficult to manage Limbus. Ideally, Limbus could be installed by the CT sim vendor, all of the contours would be run at the CT scanner and then sent directly to the export destination. Further, we have issues with the automatic recognition of structure templates based on the DICOM tags. So we just decided to apply the templates manually rather than constantly having to fix the incorrect template being chosen by Limbus. I want to see more AI integrated into clinical practice specifically for things like chart checking (initial and weekly chart checks), patient timeouts prior to treatment, daily / weekly comparison of ARIA EMR database versus third party vendors like Accuray, Elekta, etc. (For example, the treatment fraction count can mismatch between ARIA and Accuray systems; this occurs frequently, if AI could please help in searching for these mismatches.) Since some vendors have made it very difficult to use scripting with their environments (i.e. ARIA - you have to pay for scripting, Accuray - no scripting allowed), it would be cool if there was an AI "Agent" that could use the mouse and keyboard to interact with the vendor software in the event that scripting is unavailable. Or for example in Raystation an AI coding agent could help you write scripts. This would open the benefits of scripting to a wider audience. Also, if scripting could be made more portable between EMR / treatment planning systems. Currently, scripting is silo'ed into the different EMR and TPS and the scripts do not really work with one another.

**R12.** I am concerned about rapid growth without appropriate oversight in clinical implementation that may be advertised as plug and play solutions. I am also concerned about users falling into habit of trusting or deferring to a product's solution without appropriate review or knowledge of how the system arrived at its conclusion.

**R13.** not enough quality control of AI tools, not enough benchmarking of AI tools

**R14.** Sure, people blindly accept or paste the results without verifying their validity.

**R15.** It is not really at a level that if can be used make clinical decisions, but it is at the level that it can take care mundane tasks and accelerate workflows. I believe there is too much of a push to put AI in the position to make "decisions". AI should just be used alleviate clinicians of the workload that comes from having to do repetitive and some administrative tasks that take clinicians from focusing on patient care and scientific advancement. AI may be trusted to make "decisions" once researchers have developed a more solid understanding of how more complex AI work.

**R16.** I have concerns about blind implementation of AI tools by people/clinics who may not have large groups or time to understand the limitations of the technology.

**R17.** Be care

**R18.** I think AI will generally lead to the downfall of humanity. Humans need work.

**R19.** I can definitely see benefits to using AI to assist human labor in radiation oncology.

**R20.** I think there is more AI could do with respect to contouring normal anatomy. I worry that people will come to rely on it excessively in an effort to save time. Also, I tend not to trust medical soft ware when I don’t know how it was developed

**R21.** For AI contouring, there isn't a metric provided (that I'm aware of) about how confident the model feels it generated the contours. This would help with contour review or extent of editing that may be appropriate. I have concerns that we are accepting AI contours for target volumes, particularly nodal, which may be more extensive or less inclusive than self-generated ones. I feel we need to be careful to accept an "output" to directly apply to treatment planning or we may run into toxicity or treatment failures as a result of overlooking over/under contouring (targets or normal structures)

**R22.** Rigorous safeguards required to prevent PHI breaches and false answers

**R23.** I will use OpenEvidence on occasion for rare histologies or clinical scenarios. I do worry about hallucinations though.

**R24.** I would be concerned if there was not immediate oversight and physician directed evaluation of the AI input

**R25.** I worry about our dosimetrist. Eventually it will replace many of us too but I predict that is a long way off.

**R26.** Yes - it should be physician driven and very carefully implemented to supplement and support existing physicians. There is serious risk for error and patient harm if not properly utilized. There is serious risk for it being implemented in a way the improves team efficiency but then requiring us to handle larger and larger volumes to optimize $, compromising work/life balance and patient experience. Additionally, if AI becomes sufficiently advanced it could replace human jobs which would have significant social, economic, and psychological impacts

**R27.** A good solid foundation of pertinent knowledge is imperative to know when the AI tool may be wrong.

**R28.** I think it is insufficient and frequently wrong, and people rely too much on it without double checking

**R29.** It’s still in its infancy - will probably be much different and more intertwined with practice by 10 years from now

**R30.** Yes, bias in data, hallucinations, corporate interest in influencing practice

**R31.** AI chat GPT responses are 85% correct, but always confident. It could lead to errors.

**R32.** There’s a considerable amount of redundancy in the work that I do if I could help reduce that it would be a great help

**R33.** not really, as long as it is acknowledged that every process requires some form of QA before it is trusted to be correct a satisfactorily high % of the time

**R34.** For writing notes, AI tools still have a way to go before I can just click "sign."

**R35.** I use AI a lot more to help with note writing currently than I do as a clinical tool

**R36.** I think it is helpful, but it should always be cross-checked, as the algorithm may sometimes lead to misinformation or require certain keywords in the search to get the information you are looking for, in my opinion.

**R37.** Laziness and paradoxically increasing human error in certain cases

**R38.** I think the abilities of AI are oversold at present and frequent errors are made - I only use it for noncritical OARs

**R39.** The contouring tool are just now getting to the point where they do not need so much revision that it is easier to draw from scratch. I have worked for years with tools that barely resulted in any time savings but not there is definite time savings with autocontouring of node regions.

**R40.** Would welcome further integration of AI tools to reduce administrative burden in Radiation Oncology.

**R41.** We must read responses with scrutiny and verify the sources provided by AI. My current use is as a research tool and a brainstorming companion.

**R42.** not yet

*Response numbers (R1, R2, …) are sequential within the set of non-empty comments and do not correspond to respondent order in the source data.*
